# New revelations on the interplay between cardiomyocyte architecture and cardiomyocyte function in growth, health, and disease: a brief introduction

**DOI:** 10.1098/rstb.2021.0315

**Published:** 2022-11-21

**Authors:** Vijay Rajagopal, Christian Pinali, Holly A. Shiels

**Affiliations:** ^1^ Department of Biomedical Engineering, Faculty of Engineering and IT, The University of Melbourne, Victoria 3010, Australia; ^2^ Baker Department of Cardiometabolic Health, The University of Melbourne, Melbourne, VIC 3010, Australia; ^3^ Division of Cardiovascular Sciences, Faculty of Biology Medicine and Health, University of Manchester, Manchester M13 9NT, UK

Physiologic function is the consummation of a meaningful biologic event on a matrix of structure. [1, p. 19]

## 1. Introduction

The heart is a dynamic organ whose form and function change in accordance with changes in cardiovascular demand from the body. Short-term increases in demand are met almost instantaneously by increasing cardiac output. These rapid changes are driven by an intricate cellular network of chemical and mechanical signalling pathways. During longer-term changes in blood supply demand, as in growth or with disease, altered cellular chemical and mechanical signals initiate structural remodelling to reset cardiac output to the new level of demand. Thus, changes in cardiac demand which occur as we grow and age, during disease and recovery or with extreme training, are underpinned by the interplay between functional and structural remodelling of the cardiomyocyte. Additionally, cardiomyocyte function and structure differ across animal taxa, reflecting the varying cardiovascular demands that exist between, for example, invertebrates, fishes, birds and mammals. In this special issue, we offer a collection of state-of-the-art research articles, reviews and methods that investigate the intimate relationship between cardiomyocyte function and architecture. The collection emphasizes the need for, and epitomizes the success of, merging traditional sub-disciplines within cardiomyocyte biology to gain an integrated understanding of how structure and function are linked in adaptive and maladaptive cardiac remodelling. Here, we provide a short introduction to the papers contained within this special issue organized into key areas but highlight cross-talk between areas. We follow this by briefly discussing the technical advances in imaging, micro- and nanoscale manipulation, bioinformatic analyses and computational approaches that underpin many of the studies contained herein and reinforcing the link between technological development and scientific advancement.

## 2. Transverse tubule and dyadic architecture and excitation–contraction coupling

Cardiomyocyte architecture is critical for efficient cardiac muscle contraction. Every heartbeat is initiated by an electrical trigger that propagates from the sinoatrial node to the sarcolemmal membrane. The juxtaposition of voltage-activated L-type calcium channels on the transverse tubule to the ryanodine receptors on the sarcoplasmic reticulum at the cardiac dyad converts the electrical trigger into calcium-induced calcium release. The myofibrils and mitochondria that are in the immediate vicinity of the dyad use this calcium signal to initiate contraction and enhance ATP production to meet the energy demand, respectively. Mackrill [2] reconstructs the evolutionary history of the cardiac dyad, the site of communication between the sarcoplasmic reticulum and transverse tubules across metazoan. This work reveals that cardiac dyads are present in mammals, arthropods and molluscs, but not in all other animals studied to date. In mammals, the dyads have been proposed to underly the strong and fast calcium transients that underpin the high heart rates and pressure development in a heart required to sustain endothermy. Paradoxically, Shiels [3] shows that endothermic bird heart is capable of stronger and faster cardiac contraction than mammals even though bird myocytes lack dyadic architecture. Shiels' analysis discusses the organization of clusters of ryanodine receptors termed ‘calcium release units’ [4] as a compensation for the lack of dyads in the bird cardiomyocyte.

The organization of ryanodine receptor clusters is investigated at unprecedented detail, in this special issue, through the use of enhanced expansion microscopy by Sheard *et al*. [5] (and see the image on the cover of this special issue collection). The new data reveal ryanodine receptors forming frayed clusters when junctophilin is under-expressed in disease. Based on the structural evidence in their data, Sheard *et al.* propose that junctophilin not only tethers the sarcoplasmic reticulum to the transverse tubule but also has a direct role in the maintenance of ryanodine receptor clustering. This opens up questions about putative mechanisms governing ryanodine receptor clustering, and the potential for a feedback loop between dyadic cleft architecture and ryanodine receptor clustering in disease progression. Indeed, altered transverse tubule architecture and desynchronized calcium signalling were investigated in an ovine model of myocardial infarction by Perera *et al.* [6]. They show that transverse tubule fragmentation, as well as enlargement and elongation of surviving tubules, are defining characteristics of the infarct border region. This phenomenon impacts dyadic function with consequences for regional contractility of the cardiac tissue.

## 3. Cardiomyocyte architecture and calcium signalling

While ryanodine receptors expressed in cardiac dyads are primarily the site of elementary calcium release events, sarco-endoplasmic reticulum calcium pumps (SERCA) regulate calcium re-uptake into the sarcoplasmic reticulum to effect cardiomyocyte relaxation. Holmes *et al*. [7] study the effect of spatial organization of these pumps on calcium homeostasis. Using a combination of image analysis and computational modelling they show that alterations to SERCA distribution can increase or decrease the cell's vulnerability to cardiac alternans.

Inositol triphosphate receptors (IP3-receptors) are also present in the sarcoplasmic reticulum membrane of cardiomyocytes and release calcium into the cytoplasm when opened. In atria and ventricles of mammals, IP3-receptors localize with ryanodine receptors at peripheral and dyadic microdomains, respectively, and impact excitation–contraction coupling when expressed at sufficiently high levels. In non-mammalian vertebrates like birds, non-avian reptiles and fishes, where the size and structural organization of ryanodine receptor clusters are reduced, IP3-receptors may play a greater role in excitation–contraction coupling [3]. In this special issue, Demydenko *et al.* [8] reveal the myriad of ways IP3-receptors contribute to cardiomyocyte function, beyond excitation–contraction coupling, simply by the specialized microdomains they localize to. The role IP3-receptors play in coupling ion channel calcium fluxes from the sarcoplasmic reticulum into mitochondria via sarcoplasmic reticulum–mitochondria contact sites emerges as an interesting new avenue for understanding cellular energy flux. These fluxes boost ATP production by mitochondria in a beat-to-beat basis and also trigger apoptotic signalling under conditions of greater mitochondrial calcium content [8]. Demydenko *et al.* also show IP3-receptors contribute to calcium flux in the cardiac nucleus, where calcium mediates the activity of calcium-dependent transcription factors for hypertrophic gene transcription. Continuing on the theme of nuclear calcium, Voglhuber *et al.* [9] show for the first time that mitochondria residing near the nucleus modulate nuclear calcium dynamics. Perinuclear mitochondrial dysfunction leads to disproportionate rise in nucleoplasm calcium at high pacing frequencies. Together with the measured reduction in mitochondrial content in their heart failure model, their results directly point to the integral role played by the organization of cellular components in affecting calcium-dependent growth signalling.

## 4. Cardiomyocyte architecture in energetics

Several authors examine mitochondria organization and bioenergetics in relation to cardiac function and growth in this special issue. Birkedal *et al*. [10] start with a broad literature review of the structural changes cardiomyocytes undergo through development, observing that cardiomyocytes from fishes and neonatal mammals exhibit similar architectures owing to similar workloads. They then detail the discoveries that lead to our current understanding of metabolite transport within the vertebrate cardiomyocyte providing the baseline for understanding changes to cellular energetics with change in demand.

Kim *et al*. [11] present primary data on the post-natal development of mitochondrial organization. They show mitochondrial volume fraction increases 1.4-fold between neonatal and mature states, reflecting increased capacity for ATP production that is in line with increased blood supply demand at maturity. Mitochondrial surface to volume ratio is highest immediately after birth and reduces through maturation. High surface to volume ratio elevates metabolite concentrations in the cytoplasm and can compensate for reduced oxidative phosphorylation capacity [12]. Kim *et al.* further reveal greater interactions between mitochondria and lipid droplets in the early stages of post-natal development. These lipid droplet-associated mitochondria all had significantly larger inter-mitochondrial junctions.

Morphological and organizational changes in mitochondria have long been observed in cardiac remodelling. We have yet to find a way to precisely understand the mechanisms that are behind these structure–function associations. This is because we are always faced with a chicken–egg question: is the structural change a cause of growth/disease or an effect of growth/disease? Similar to Holmes *et al*. [7] with regard to calcium signalling, Ghosh *et al*. [13] offer a possible solution to the chicken-egg question by creating an *in silico* model that integrates electron microscopy data with independent mitochondrial functional measurements in the literature to predict the explicit effects of structural disorganization on bioenergetics. With their model, they find that mitochondrial organization affects the homogeneity of energy supply across the cardiac myocyte.

## 5. Cardiomyocyte architecture and mechanosensing

All cells, but particularly cardiomyocytes, sense and respond to mechanical signals in their environment. The cytoskeleton is the structure upon which much of the cell architecture is built and is vital for sensing and transducing mechanical signals between organelles, and between the cell and the extracellular environment. In this issue, Hawkes *et al*. [14] delve deeply into the role of transmembrane integrins, proteins which connect ligands in the extracellular matrix (e.g. laminin and fibronectin) with the cytoskeleton. By varying the combinations of ligand and integrin and testing their impact on adhesion formation, traction and mechanosensing, they were able to define their singular and combined role in cardiomyocyte adhesion formation and mechanosensing. This study involved the use of DNA origami to control the spacing of the ligand–integrin interactions to the nanoscale and thus offer novel perspective on global versus nanoscale adhesion interactions on mechanosensing. The cytoskeleton does more than control cellular mechanosensing. It also plays a role in regulating cellular energetics. Solomon *et al*. [15] in this special issue show that the cytoskeleton is essential for cellular energetics, and show that disrupting the interaction between mitochondria and the cytoskeletal network is associated with poor energy balance in heart failure and cancer.

The culmination of cardiomyocyte excitation–contraction coupling is the contraction of the cell which helps the heart fulfil its role of pumping blood around the body. The myofilament apparatus, and its single contractile unit, the sarcomere, are thus the end-effector of many of the cardiomyocyte signalling pathways discussed thus far. Ahmed *et al.* [16] present a new perspective on the sarcomere as a regulator of organelles within the cell, in particular the mitochondria. They first review the mechanisms that regulate sarcomeric function during maturation, and then integrate this with the interplay between the sarcomere, the mitochondria, the sarcoplasmic reticulum, transverse-tubules and the nucleus during maturation to show a tight dance where each party works together for healthy maturation and to show how disease can occur when these interactions go wrong.

A further step towards the understanding of cardiomyocyte mechanics and mechanosensing is accomplished by Peyronnet *et al.* [17]. Using a combination of atomic force microscopy, which probes the surface of a cell obtaining topographical and force/stiffness information, and carbon-fibre-based axial stretch [18], they are able to separate the impact of indentation forces and longitudinal forces on function in a beating cardiomyocyte.

Brayson & Shanahan [19] provide intriguing novel insight into spatial re-localization of lamin A, which, before moving to the nucleus in its mature form, is localized in the sarcomere in its immature form as prelamin A. Accumulation of immature lamin A in the sarcomere seems to point towards sarcomere dysfunction rather than nucleus rupture as a possible cause of farnesylated lamin A cardiomyopathy.

## 6. Technological innovations underpin our understanding of cardiomyocyte structure–function biology

The significant advances in our understanding of cardiomyocyte architecture and function have been made possible by technological innovation. Major technical innovations presented in this special issue include advanced three-dimensional microscopy techniques, artificial intelligence for image segmentation, and data-driven biophysics-based computational modelling.

Microscopy is the first tool used to understand the structure of cells. Confocal microscopy has become the go-to tool to investigate the structure of the most prominent organelles and proteins in the cardiomyocyte. It combines ease and speed of sample preparation with a vast array of antibodies and voltage-sensitive dyes, to provide volumetric imaging and functional assessment of features of interest within a cell. Typical resolutions are about 180 nm in the confocal plane and about 500 nm in the axial direction [20]. Super-resolution microscopy and electron microscopy (EM) overcome the resolution limitations of light microscopy, with section thickness being the ultimate limitation of three-dimensional electron tomography [21,22]. This problem is overcome by scanning volumetric microscopy which, in its variants of focused ion beam scanning electron microscopy (FIB-SEM) and serial block face scanning electron microscopy (SBF-SEM), allow for serial imaging of large (4 × 4 × 4 µm^3^) to (50 × 50 × 50 µm^3^) volumes of tissue with planar resolution approximately 10 nm and *z*-resolution 50–10 nm. In this issue, Perera *et al.* [6] used SBF-SEM to understand the regional variation of transverse-tubule remodelling in cardiac failure following myocardial infarction in a sheep model of disease. Kim *et al.* [12] use FIB-SEM to understand how the architectural interplay between lipid droplets, mitochondria, the sarco-tubular system and the contractile apparatus, impact energy distribution within the cardiomyocyte during postnatal development.

A typical microscope modifies light paths to provide a magnified view of a cell's contents. However, what if we could make the cell itself larger? This is the radical but effective approach of enhanced expansion microscopy where a tissue or isolated cells are embedded in a gel which expands after polymerization causing a dilation up to 10 times of the tissue studied. Expansion combined with confocal microscopy increases the effective resolution. Sheard *et al*. [5] used enhanced expansion microscopy to observe topological complexities, geometries and molecular sub-domains within ryanodine receptor clusters in health and disease in myocytes.

Both light and electron microscopy have gone from being low throughput two-dimensional image acquisition technologies to big-data generators. This evolution of data collection has demanded a parallel evolution in the high throughput data processing. Big datasets produced by three-dimensional electron microscopy/EM require the application of artificial intelligence pipelines like CardioVinci by Khadangi *et al.* [23] as well as broadly applied software like ImageJ and DragonFly. The special issue also includes the work of Frisk *et al.* [24] who have developed ‘Tubulator’ a stand-alone image analysis tool written in Matlab for the measurement of transverse tubule distances to the sarcolemma and distances between ion channels in isolated cells and tissue sections. This package is also designed to help overcome the data analysis bottleneck of confocal imaging.

High resolution microscopy and microscopy image segmentation tools now enable the creation of ultrastructurally detailed computational models of cardiomyocyte form and function as shown by Holmes *et al*. [7] and Ghosh *et al*. [12]. In both studies, we see that spatially detailed models help interpret the geometric information in structural microscopy data. By using these models to isolate and change specific structural features or functional components we are able to quantitate the sensitivitiy of cardiomyocyte function to changes in its form. Microscopists inherently understand the importance of structural architecture for function as eloquently expressed in the quote by JR Sommer [1] that heads this introduction.

## 7. Concluding remarks

Indeed, an integrated understanding of cardiomyocyte form and function is required to progress cardiac health and disease research. However, the majority of work in cellular cardiology over the past 50 years has been carried out in fairly siloed research fields. Thus this all important integration of form and function has remained elusive. The collection of papers contained within this special issue was ensembled to make a step change. New approaches for quantifying changes in cellular architecture have been presented and paired with computational integration to allow extrapolation across species, maturation and across health and disease. By linking overarching regulators and end-effectors like calcium, the sarcomere and energetics, with novel structural imaging techniques which allow us to place these key players across the myocyte in space and time, the integration and visualization of structure and function begin to emerge. May the image get ever clearer.

## Data Availability

This article has no additional data.
